# *NTRK1* Fusion in Glioblastoma Multiforme

**DOI:** 10.1371/journal.pone.0091940

**Published:** 2014-03-19

**Authors:** Jinkuk Kim, Yeri Lee, Hee-Jin Cho, Young-Eun Lee, Jaeyeol An, Gye-Hyun Cho, Young-Hyeh Ko, Kyeung Min Joo, Do-Hyun Nam

**Affiliations:** 1 Samsung Biomedical Research Institute, Samsung Medical Center, Seoul, Korea; 2 Institute for Refractory Cancer Research, Samsung Medical Center, Seoul, Korea; 3 Samsung Advanced Institute of Technology, Samsung Electronics Co., Ltd., Seoul, Korea; 4 Graduate School of Health Science & Technology, Samsung Advanced Institute for Health Science & Technology, Seoul, Korea; 5 Department of Pathology, School of Medicine, Sungkyunkwan University, Seoul, Korea; 6 Department of Anatomy and Cell Biology, School of Medicine, Sungkyunkwan University, Seoul, Korea; 7 Department of Neurosurgery, School of Medicine, Sungkyunkwan University, Seoul, Korea; The Ohio State University Medical Center, United States of America

## Abstract

Glioblastoma multiforme (GBM) is the most aggressive form of brain tumor, yet with no targeted therapy with substantial survival benefit. Recent studies on solid tumors showed that fusion genes often play driver roles and are promising targets for pharmaceutical intervention. To survey potential fusion genes in GBMs, we analysed RNA-Seq data from 162 GBM patients available through The Cancer Genome Atlas (TCGA), and found that 3′ exons of neurotrophic tyrosine kinase receptor type 1 (*NTRK1*, encoding TrkA) are fused to 5′ exons of the genes that are highly expressed in neuronal tissues, neurofascin (*NFASC*) and brevican (*BCAN*). The fusions preserved both the transmembrane and kinase domains of *NTRK1* in frame. *NTRK1* is a mediator of the pro-survival signaling of nerve growth factor (NGF) and is a known oncogene, found commonly altered in human cancer. While GBMs largely lacked *NTRK1* expression, the fusion-positive GBMs expressed fusion transcripts in high abundance, and showed elevated *NTRK1*-pathway activity. Lentiviral transduction of the *NFASC-NTRK1* fusion gene in NIH 3T3 cells increased proliferation *in vitro*, colony formation in soft agar, and tumor formation in mice, suggesting the possibility that the fusion contributed to the initiation or maintenance of the fusion-positive GBMs, and therefore may be a rational drug target.

## Introduction

Glioblastoma multiforme (GBM) is the most common and aggressive form of brain tumor. The current established first-line therapy––surgical resection and adjuvant chemoradiotherapy with temozolomide––provides mostly palliation, and thus the five year survival rate of GBM patients is only ∼10% [1]. Unfortunately, attempts to target well-known molecular aberrations of GBMs, such as *EGFR* and *MET*, have so far been only marginally successful [1]. Therefore, there is an urgent need to identify novel molecular targets that can be critical for GBM initiation and progression.

Gene fusion has been known to be critical cancer driver mutation in hematopoetic-origin tumors [2]. Recent studies have shown that these fusion events also occur in various types of solid tumors [3]–[6]. Many of the recent discoveries of the fusions were powered by massively parallel sequencing of cancer genome, exome, or transcriptome [5], [6]. Especially with the availability of large-scale sequencing data from public cancer sequencing projects, such as The Cancer Genome Atlas (TCGA) [7], the discovery of novel fusion genes or other important alteration events is becoming increasingly dependent on the use of sophisticated analytic strategies.

Here, we describe our independent survey of gene fusions in GBMs with the TCGA data, and follow-up experiments with the *NFASC*-*NTRK1* fusion gene. We show that NIH 3T3 cells that express the *NTRK1*-fusion are tumorigenic in nude mice, suggesting that the possibility that the fusion played similar driver role during the initiation or progression of the fusion-bearing GBMs.

## Results

### Survey of Gene Fusions in GBMs

To search for novel gene fusions in GBM, we analyzed paired-end RNA-Seq data of 162 GBM patients, available through TCGA [7]. A subset of modules from the FusionSeq pipeline [8] was adopted to score potential fusions based on the number of “discordant read pairs.” The discordant read pairs are paired-end reads whose 5′ and 3′ reads map to different genes, thereby supporting the fusion between the genes. The number of discordant read pairs was then normalized by the gene expression level of the two involved genes, since higher expression could proportionally increase false-positive discordant pairs.

Among the 20 fusions that scored highest by the analysis, *FGFR3-TACC3* was found in two independent samples; one (Sample ID, 1835) with the highest score and the other (Sample ID, 4925) with the 15^th^ highest score (Table 1, Table S1 and S2). Because *FGFR3-TACC3* was recently reported as a key oncogenic driver in the GBMs that harbor the fusion [6], our rediscovery of this fusion confirmed the validity of our analytic approach. Other candidates in the top-20 candidate fusions included ones that involved *EGFR* and genes within 1 Mb radius of the EGFR locus (*CO9, VSTM2A, LANCL2, PSPH, SEPT14*, and *SEC61G*; Table 1). All the samples with those fusions were found to have high level (6–12 folds) *EGFR* gene amplification (data not shown). Because of a previous study suggesting that gene fusions associated with recurrent amplicons are by-products of chromosomal amplification and are likely passenger events [9], the *EGFR*-fusions were not followed up further. Another candidate fusion in the list, *KIF5A-BC033961* (in four samples), was also excluded, because it could be a false-positives fusion resulting from misannotation; according to ESTs, *BC033961* is a 3′ part of *KIF5A*. Among the remaining 7 candidates, four were further filtered out during the downstream analyses for reasons indicated in Table 1. The final three candidates included two fusions that involved neurotrophic tyrosine kinase receptor type 1 (*NTRK1*, encoding TrkA), and a fusion between *YEATS4* and *XRCC6BP1* (Figure S1). Three recent independent studies (which came out while this work was in review) also reported evidences consistent with the presence of *NTRK1*-fusions in the TCGA GBM panel [10]–[12].

**Table 1:** Top-20 potential gene fusions predicted by discordant read pair analysis.

aFusion Type	bGene1	bGene2	cSample ID	dScore (RESPER)	Reason for Exclusion
**read-through**	***FGFR3***	***TACC3***	**1835**	**108.2**	
intra	*EGFR*	*CO9*	5209	89.3	EGFR amplicon
read-through	*KIF5A*	*BC033961*	0618	74.5	Misannotation
intra	*DTX3*	*FRS2*	2571	74.0	Off-frame
read-through	*VSTM2A*	*EGFR*	0747	72.9	EGFR amplicon
read-through	*EGFR*	*LANCL2*	0211	67.5	EGFR amplicon
intra	*AK293540*	*AGAP3*	2528	63.7	Off-frame
cis	*LANCL2*	*PSPH*	0817	52.8	EGFR amplicon
**intra**	***BCAN***	***NTRK1***	**2619**	**50.7**	
cis	*LANCL2*	*SEPT14*	0211	48.6	EGFR amplicon
cis	*SEC61G*	*EGFR*	2554	45.5	EGFR amplicon
intra	*SDK1*	*EGFR*	2557	38.3	EGFR amplicon
intra	*MARCH9*	*SUDS3*	5856	36.6	No junction reads
read-through	*KIF5A*	*BC033961*	1980	34.9	Misannotation
**read-through**	***FGFR3***	***TACC3***	**4925**	**32.4**	
cis	*OS9*	*C12orf66*	0174	31.8	Off-frame
**intra**	***NFASC***	***NTRK1***	**5411**	**31.7**	
read-through	*KIF5A*	*BC033961*	5651	30.4	Misannotation
**intra**	***YEATS4***	***XRCC6BP1***	**0138**	**30.4**	
read-through	*KIF5A*	*BC033961*	2558	29.3	Misannotation

a Fusion type: intra, intra-chromosomal; inter, inter-chromosomal; read-through, the involved genes are adjacent and on the same strand; cis, the involved genes are adjacent and on the opposite strands.

b For the fusions that were not excluded by indicated reasons, gene 1 and gene 2 correspond to the 5′- or 3′-partner of each fusion.

c Sample IDs are abbreviated.

d RESPER is FusionSeq-reported scores for prioritization. The fusions that were not excluded are indicated in bold font.

We focused on the *NTRK1*-fusions, as they are recurrently found in two independent patients. *NTRK1* was fused with brevican (*BCAN*) in one patient (Sample ID, 2619) and with neurofascin (*NFASC*) in another (Sample ID, 5411). *NTRK1*––encoding a receptor for NGF––is a particularly likely candidate for an oncogenic driver gene of GBM, because it positively regulates cell survival through the MAPK cascade [13], and is commonly altered in many types of human cancers, including neuroblastoma [14] and prostate [15], colon [16], thyroid [17], and lung [18] cancer. Notably, fusions associating this gene were previously discovered in colon [16], papillary thyroid [17], and lung [18] cancer. In up to 12% of papillary thyroid cancer patients, the 3′ exons of *NTRK1* with the kinase domain had been found to fuse with the 5′ exons of various thyroid-expressed genes (*TPM3, TPR, TFG*) in frame. The fusion genes were reported to contribute to the initiation and maintenance of the cancers [17].

### Structure of *NTRK1* Fusion Genes

**Figure 1:**
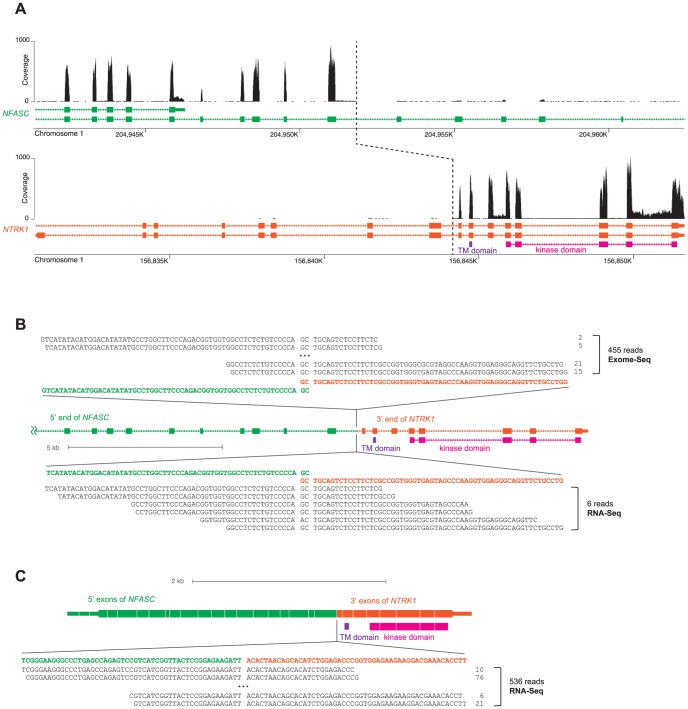
*NFASC-NTRK1* fusion. (**A**) Per-nucleotide read coverage (expression) of genomic regions along *NFASC* and *NTRK1*. The dotted line marks the DNA-level break-points in the two genes, as instructed by the fusion-point mapping result in panel B. (**B**) A schematic of pre-mRNAs of the *NFASC-NTRK1* fusion gene. Top and bottom sequences in black are the reads that map onto the DNA-level fusion-point. The fusion-point is mapped with slight ambiguity due to 2-nt-long micro-homology between the two break-points in the involved genes. (**C**) A schematic of spliced transcripts of the fusion gene. Bottom sequences in black are the reads that map onto the chimeric exon-exon splicing junction.

We analyzed the detailed structure of the two *NTRK1*-fusions. Plotting per-nucleotide read coverage of genomic regions along *NFASC* and *NTRK1* revealed abrupt discontinuation of the coverage (Figure 1A), which suggested that the 5′ end of *NFASC* is fused to the 3′ end of *NTRK1*. A more direct evidence of the fusion would be the sequencing reads that map onto the very position where the two genes were physically broken and fused together at the DNA-level. We recovered six such reads (Figure 1B). The number of reads was relatively small because the fusion point was in an intron; only a minority of the RNAs captured by RNA-Seq is the intron-containing pre-mRNAs. Such intronic reads are also uncommon in Exome-Seq, because Exome-Seq is primarily capturing exons, but not introns. However, we fortunately found >400 reads that map on the exact fusion point in the Exome-Seq data (Figure 1B), because the intronic region was fortuitously captured along with the adjacent exons. Such Exome-Seq reads were found only in the tumor tissue, but not in the blood of the same patient, indicating that the *NFASC-NTRK1* fusion occurred somatically at the DNA-level. Furthermore, we identified >500 reads, from the RNA-Seq data, that map onto the chimeric exon-exon junction of the spliced fusion transcript (Figure 1C). The fusion transcript retained the *NTRK1* transmembrane and kinase domains in frame.

**Figure 2:**
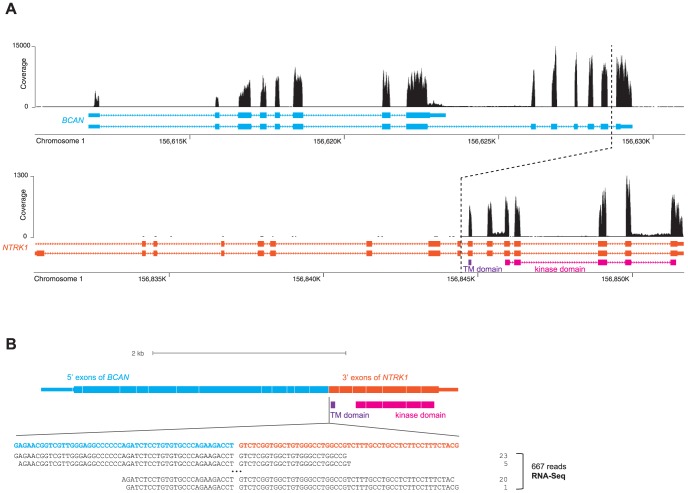
*BCAN-NTRK1* fusion. (**A**) Per-nucleotide read coverage of genomic regions along *BCAN* and *NTRK1*. The dotted line marks approximate positions where the fusion has occurred. (**B**) A schematic of spliced transcripts of the fusion gene. Bottom sequences in black are the reads that map onto the chimeric exon-exon splicing junction.

We also identified analogous *BCAN-NTRK1* fusion transcripts that retain the *NTRK1* transmembrane and kinase domains in frame (Figure 2A and 2B). However, the reads on the exact fusion point were not identified either from the Exome-Seq or RNA-Seq data. Whether *BCAN-NTRK1* fusion occurred at the DNA- or RNA-level needs to be determined when the genomic DNA of the sample (Sample ID, 2619) could be accessed.

### Molecular Consequences of *NTRK1* Fusions

**Figure 3:**
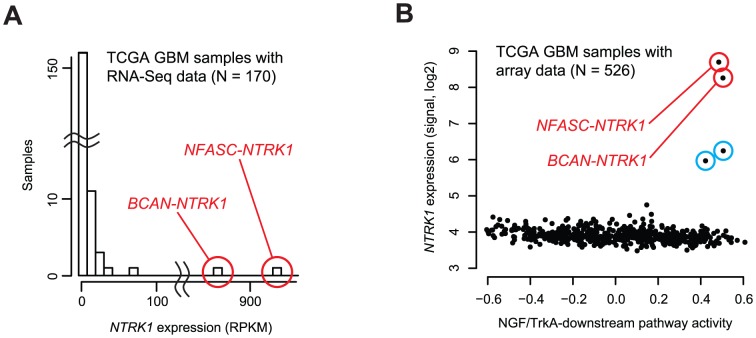
Molecular consequences of *NTRK1*-fusion. (**A**) *NTRK1* expression in 170 TCGA GBM samples (from 162 patients) with RNA-Seq data. Samples bearing *NTRK1*-fusion genes are marked and labeled. (**B**) Relationship between *NTRK1* expression and NGF/TrkA-downstream pathway activity in 526 TCGA GBM samples (from 526 patients) with microarray gene expression data. Samples with *NTRK1*-fusion are marked with red circles. Two other samples with outlier *NTRK1* expression are marked with blue circles (TCGA-32-4209, TCGA-19-5947).

Both *NFASC* and *BCAN* have been known to mediate neuronal functions [19]–[21] and were highly expressed in tissues of neuronal lineages (Figure S2). Their expression was also detected in GBMs (Figure S3). In contrast, *NTRK1* expression was essentially undetectable (above background) in the great majority of the 170 TCGA GBMs (for 162 patients). Exceptions were the two GBMs with the *NTRK1*-fusion that showed remarkably strong expression of *NTRK1* (Figure 3A). The exclusive association of the fusion with the outlier expression suggested the hypothesis that switching of the promoter of *NTRK1* with those of neuronally expressed genes causes the outlier expression of *NTRK1*, as previously observed in other fusions [22].

The pattern of *NTRK1* outlier expression in the fusion-positive samples may facilitate additional identification of *NTRK1*-fusions. Although 162 TCGA GBM cases with RNA-Seq data were subjected in this study, the TCGA GBM panel included hundreds more samples without RNA-Seq data, yet with microarray-based expression data. Among these, we identified two samples with outlier expression of *NTRK1* (Figure 3B, blue circles). Future RNA-Seq analyses on these two samples might reveal additional *NTRK1*-fusions. All four TCGA GBM samples with the outlier *NTRK1* expression showed elevated activity of the NGF/TrkA-downstream pathway (Figure 3B, Table S3), indicating that the *NTRK1* fusion gene expression in these samples had the effects consistent with the NGF-triggered activation of the NGF/TrkA-downstream pathway.

### Tumorigenic Activities of *NFASC-NTRK1* Fusion

**Figure 4:**
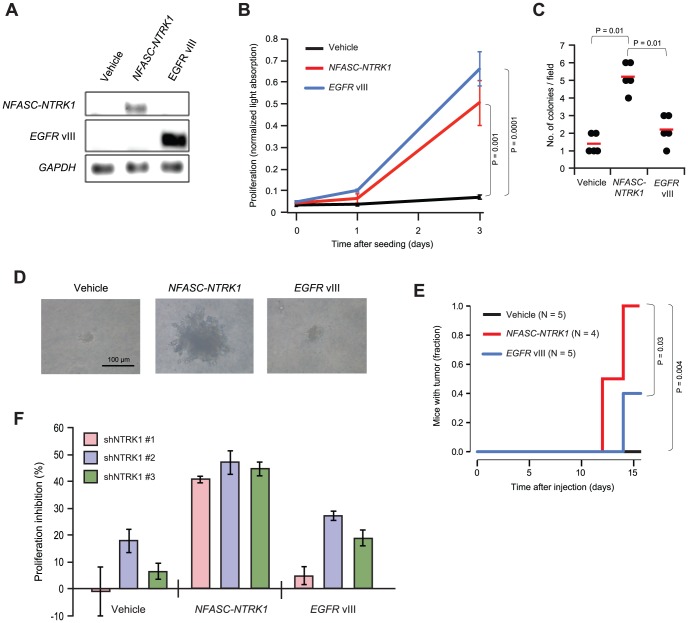
Tumorigenic activities of *NFASC-NTRK1* fusion gene. (**A**) *NFASC-NTRK1* and *EGFR* vIII mRNA expression in NIH 3T3 cells, determined by RT-PCR. (**B**) Proliferation of NIH 3T3 cells lentivirally infected with the indicated viruses. Error bars are 95% confidence intervals. (**C**) Number of colonies in a unit microscopic field, formed by NIH 3T3 cells infected with the indicated viruses. Red lines are the average within each group. (**D**) Morphology of individual colonies in soft agar, formed by NIH 3T3 cells infected with the indicated viruses. (**E**) Incidences of subcutaneous tumor formation in the mice injected with NIH 3T3 cells infected with the indicated viruses. (**F**) Inhibition of proliferation by three independent shRNAs targeting the *NTRK1* fusion transcripts. Error bars are standard deviations of five-replicate experiments.

To examine functional consequences of the *NTRK1*-fusions, we introduced the *NFASC-NTRK1* fusion gene, the *EGFR* vIII (positive control), or an empty construct (negative control) into NIH 3T3 cells––cells commonly used to assess oncogenic potential of novel oncogenes. The expression of the *NTRK1* fusion gene and the *EGFR* vIII was confirmed by RT-PCR (Figure 4A). The cells expressing the *NTRK1* fusion gene appeared to have increased phosphorylation of TrkA, but failed to show increased phosphorylation of AKT or ERK, indicating that the downstream signaling of the *NTRK1* fusion gene bypass these signaling nodes (Figure S4). The bypassing of AKT and ERK signaling nodes was also observed in a previous study of *FGFR3-TACC3* fusion gene [6]. The cells expressing the *NTRK1* fusion gene or the *EGFR* vIII proliferated significantly faster than the negative control (P = 0.001, T test; Figure 4B). On soft agar assay, the fusion gene-infected cells formed more colonies than did both the positive and negative controls (P = 0.01 for both, rank sum test; Figure 4C). Morphological examination of each individual colonies showed that the colonies formed by the fusion gene are bigger and more invasive than the colonies of both controls (Figure 4D). Furthermore, when the cells were subcutaneously injected into BALB/c nude mice, the mice injected with the fusion-gene-infected cells formed visible mass significantly faster than did both the positive and negative controls (P = 0.03 and 0.004, respectively, log rank test; Figure 4E). Together, our experimental results indicated the *NFASC-NTRK1* fusion gene confers tumorigenic function in NIH 3T3 cells, which further suggests that the fusion gene might have played driver role during the initiation or progression of the fusion-positive GBMs.

Next, we investigated whether the inhibition of the *NTRK1* fusion gene can rescue the hyper-proliferation phenotype of NIH 3T3 cells transduced with the *NTRK1* fusion gene. Three independent short hairpin RNAs (shRNAs) that were designed to target the *NTRK1*-fusion transcripts were consistently able to reduce proliferation in the fusion gene-infected cells (Figure 4F), suggesting that these NIH 3T3 cells can be utilized for identifying compounds that can effectively inhibit the *NTRK1* fusion genes. Such compounds may have potential to be developed into specific therapeutic agents for the fusion-positive patients. Thus, we tested three commercially available compounds with documented activity against TrkA (AZ-23 [23], GW441756 [24], and CEP-701 [15]) using the NIH 3T3 model. However, none showed clear effect of blocking the fusion gene-induced proliferation (data now shown). Despite the lack of efficacy of the three tested inhibitors, more comprehensive screening of larger compound libraries might lead to the successful identification of effective inhibitors of the *NTRK1* fusion gene.

## Discussion

Fusion of kinases to genes that are highly expressed in tumor or in the tissue of tumor-origin is a recurring mechanism by which tumors achieve overexpression of oncogenic kinases [2], [3], [5], [17]. As tumors with those fusions usually show strong oncogenic addiction to the fusion gene, specific inhibitors against some of these fusion genes had become the primary treatment option for the fusion-positive patients [2], [25]. To translate our finding of the *NTRK1* fusion into improved patient care, effective inhibitors of the *NTRK1* fusion genes first need to be identified. Then, oncogenic dependency of the *NTRK1* fusion-positive tumors to the fusion gene should be demonstrated. Examining genetic alterations of major GBM driver genes in the two TCGA GBM tumors with the *NTRK1* fusion (Figure S5), we found that the *NTRK1* fusion accompanied mild amplification (<2 folds) of *EGFR*. As the amplified *EGFR* may mediate resistance to *NTRK1* inhibition therapy [26], combinational use of *NTRK1* and *EGFR* inhibitors may need to be explored. Experimental validation of such targeting strategies would require models (such as glioma stem cells or xenografts [27]) derived from patient tumors with the *NTRK1*-fusion.

The involvement of *NTRK1* in GBM has been largely unknown, but *NTRK1* is involved in other types of cancer [14]–[18]. In particular, *NTRK1* has been found to fuse with tropomyosin in colon cancer [16], *TPM3*, *TPR*, and *TFG* in thyroid cancer [17], and *MPRIP* and *CD74* in lung cancer [18]. Among the partner genes of *NTRK1*, most, with the exception of *CD74*, harbor coiled-coil domains, which were shown to mediate dimerization of the fusion genes and consequent activation of the TrkA kinase domain. *NFASC* and *BCAN* are two more exceptions for lacking coiled-coil domains. Instead of coiled-coil domains, Ig-like domains appear to mediate dimerization of TrkA. TrkA has Ig-like domains within the extracellular portion of the protein, which mediate NGF-dependent dimerization [28]. A previous study showed that, if the Ig-like domains of TrkA were switched with Ig-like domains of c-Kit, the foreign Ig-like domains then mediate NGF-independent, constitutive dimerization of TrkA [28]. As *NFASC* and *BCAN* contain Ig-like domains, fusion of *NTRK1* with these partner genes results in the switching of the Ig-like domains of *NTRK1* with those of the partner genes (Figure S6). It is possible that the foreign Ig-like domains in the fusion protein mediate constitutive dimerization and activation of the TrkA kinase domain.

Members of the receptor tyrosine kinase gene family including *EGFR, MET, PDGFRA*, and *FGFR3* has been known to be heavily involved in the initiation and progression of GBM [1], [6], [7]. Our results implicate yet another member of the family, *NTRK1*, to GBM, allowing to account for the mechanism of an additional meaningful fraction of GBMs. A recent study reported an oncogenic fusion of *EGFR-SEPT14* in the TCGA GBM panel [11]. This fusion was identified as the top 55^th^ candidate by our analysis (Table S1). *EGFR-SEPT14*, which resides in a recurrent amplicon, is an exception to the previously reported trend that fusions in association with recurrent amplicons are commonly passenger events [9]. The identification of these new fusions helps reinforce the notion that GBM is a remarkably heterogeneous disease in its genetic basis, and emphasizes the need for genomic diagnostic approaches for individualized treatments.

Our discovery of *NTRK1*-fusion in ∼1% of GBMs (2 out of 162 patients) adds to the previous discovery of *FGFR*-fusion in ∼3% of GBMs [6] and *EGFR*-fusion in ∼4% of GBMs [11], tallying the GBMs with a driver fusion up to ∼8%. We envision that these may be just a few of many more driver fusions to be discovered in GBMs––albeit each would be at low-frequency––by future analyses of a larger number of GBM samples with more sophisticated technologies.

## Materials and Methods

### Discordant Read Pair Analysis

Access to TCGA controlled-access data was approved by the TCGA data access committee (Institution: Samsung Medical Center, PI: D.N.). 170 RNA-Seq data files in BAM format (for 162 GBM patients) were downloaded from CGHub. Picard was used to convert the BAM files into paired-end FASTQ files, with length of each sequence being 76 nucleotide (nt). In order to increase the distance between the two ends of a pair––and thereby increase the chance of capturing the fusion-point within the distance, the 76-nt reads were trimmed to leave 5′-most 30 nucleotides. The trimmed 30-nt reads were saved into separate paired-end FASTQ files. GSNAP [29] was used to perform paired-end-mode mapping of the trimmed reads onto hg19, without allowing any mismatch, indel, or splicing within a read. The GSNAP alignments––formatted as SAM files––were sorted so that two reads of each pair is in order of chromosomal location. The resulting SAM files were converted into MRF format using RSEQtools. With the MRF files as input, the FusionSeq (version 0.6.1) [8] pipeline was run with default parameters, until the part that calculates confidence estimates for fusion candidates. “Abnormal insert size filter” and “small homology filter” modules in the pipeline were not used, because the modules filtered out the true positive fusion of FGFR3-TACC3 [6]. All the 170 result files were merged, filtered (DASPER >1.0, RESPER >1.0, as suggested in Sboner *et al*. [8]) and sorted by RESPER (in descending order; Table S1 and S2). Each of the top-20 candidates was examined with the analyses described in the subsequent sections. For the four *NTRK1*-outlier samples (TCGA-19-2619, TCGA-06-5411, TCGA-32-4209, TCGA-19-5947), Exome-Seq data were downloaded and processed through the discordant read pair analysis pipeline, as were RNA-Seq data, except the raw predictions from FusionSeq were not subject to any filtering module. From the unfiltered prediction results, the UCSC Known Gene IDs for *NTRK1* were searched for.

### Per-nucleotide Coverage Analysis

The GSNAP-mapped SAM files (from RNA-Seq data) from the discordant read pair analysis were converted into BAM files by SAMtools, and then into BED files by bamToBed of bedTools. The BED files were sorted by chromosomes, and then converted into bedGraph files by using genomeCoverageBed of bedTools. The bedGraph files were finally converted into bigWig files by using bedGraphToBigWig, before being loaded into the UCSC genome browser for visual examination of exonic expression.

### Chimeric Splicing Junction Analysis

For each fusion candidate of interest, the sequence (including exons, introns, and 1-kb flanking regions) of the two involved genes were downloaded from the UCSC genome browser. Using the gene sequences as references, GSNAP was used to perform single-end-mode, splicing-allowed mapping of all original untrimmed (76 nt) RNA-Seq reads of the sample harboring the potential fusion. The mapping results were filtered to retain only the reads that aligned at the chimeric exon-exon splicing junction bridging the two genes.

### Break-/fusion-point Mapping Analysis

For samples with fusion candidates of interest, all original 76-nt reads (from tumor RNA-Seq data and tumor/normal Exome-Seq data) were trimmed to leave 17-nts from each side. The resulting pairs of 17-nt reads were saved as paired-end FASTQ files. GSNAP was used to perform paired-end-mode mapping of the reads on the pair of gene sequences involved in the fusion, without allowing any mismatch, indel or splicing. Subsequent filters were applied to the results to identify the paired-end reads that satisfy the following criteria: (1) each end aligns to different genes, (2) either end aligns to the intron where a break-point is suspected to be found based on the exon expression profile from the per-nucleotide coverage analysis, (3) neither end aligns to RepeatMasker-annotated regions. The identified paired-end reads were used to track down the original 76-nt reads from which the paired-end reads were derived. Finally, the identified 76-nt reads were mapped on hg19 by BLAT to reveal break-/fusion-points.

### Transcript Quantity Analysis

DEGSeq was used to calculate RPKM from the hg19 refFlat file downloaded from the UCSC genome browser and the BED files that were generated during the per-nucleotide coverage analysis.

### Microarray-based Gene Expression and Pathway Activity Analyses

Fully processed (level III) gene expression data (platform: Affymetrix U133A Plus 2.0 arrays) for 526 GBM patients (along with the expression data for 10 unmatched normal brain tissues) were downloaded from the TCGA data portal website. Because all *NTRK1*-specific probes of the Affymetrix array are targeting last three exons of *NTRK1* (which are retained in the fusion transcripts), the *NTRK1*-specific probes can detect fusion gene expression. For calculation of NGF/TrkA-downstream pathway activity, the NGF/TrkA pathway signature was defined: for the signature, we utilized microarray datasets from a previously published study (GSE18409). In this study, gene expression was measured 24 hours post NGF treatment on *NTRK1*-expressing cells; the cells were originally *NTRK1*-negative human neuroblastoma cell line SH-SY5Y, but modified to express *NTRK1*. We compared the expression profile (GSM459015) to the average profile of mock-treated cells (GSM458998, GSM459011, GSM459024, GSM459025) to calculate differentially expressed genes (150 genes; cutoff, 2 fold), which was defined as the NGF/TrkA pathway signature (Table S3). The signature did not include *NTRK1*. The signature was applied to the gene expression profile of each GBM sample in TCGA datasets, to calculate cosine-similarity between the profile and the signature using the Nearest Template Prediction [30] implemented in GenePattern. The similarity––ranging from −1 to 1––was defined as the pathway activity, with values bigger than 0 indicating relative activation, and smaller than 0 indicating relative inactivation. For examining the expression of *NTRK1, NFASC*, and *BCAN* in a panel of normal human tissues, a preprocessed microarray expression dataset was downloaded from the official website of the gene atlas project [21].

### Cell Culture

NIH 3T3 cells were obtained from the Korean Cell Line Bank, and expanded in DMEM supplemented with the final concentration of 10% BCS, 100 units/ml penicillin, and 100 μg/ml streptomycin.

### Lentivirus Production and Infection

A copy of the *NFASC-NTRK1* fusion gene was synthesized by Bioneer, and then PCR-amplified. *EGFR* vIII was RT-PCR-amplified from a GBM patient sample, and the gene sequence was confirmed as an exon-2–7-deleted form of an *EGFR* RefSeq entry, NM_005228.3. The *NFASC-NTRK1* and *EGFR* vIII fragments were subcloned into an entry vector using pENTR™/D-TOPO® Cloning Kit (Invitrogen), and then transferred into pLenti6.3/V5-DEST plasmid using LR clonase reactions (Invitrogen). The lentiviral vectors (pLKO.1-puro) harboring shRNAs targeting *NTRK1* were purchased (Thermo). The targeting sequences of the shRNAs were within the exons of *NTRK1* that were retained in the *NFASC-NTRK1* fusion transcript, and the sequences (anti-sense to the *NTRK1* mRNA) are as follows: shNTRK1 #1, 5′-TAATAGTCGGTGCTGTAGATA; shNTRK1 #2, 5′-TAGATATCCCTGCTCATGCCA; shNTRK1 #3, 5′-AAGTATTGTGGGTTCTCGATG. To generate lentiviruses, the lentiviral plasmids were cotransfected with the packaging vectors (VSVG and PAX2) into HEK293FT cells (Invitrogen) using Lipofectamine 2000 (Invitrogen). After transfection, media supernatants were collected for three days with an interval of 24 hours. For infection, NIH 3T3 cells were incubated with the lentivirus supernatants (without dilution) for 2 days. After replating, the cells were subjected to selection with blasticidin (4 μg/ml; for pLenti6.3/V5-DEST) or puromycin (3 μg/ml; for pLKO.1-puro) for 3 days.

### RT-PCR

RNeasy Mini Kit (Qiagen) was used to isolate RNAs. SuperScript III First-strand cDNA synthesis kit (Invitrogen) was used to synthesize cDNAs from the RNAs. 30 cycles of PCR were performed with the following primers: 5′-TGCACCACCAACTGCTTAG (forward) and 5′-AGAGGCAGGGATGATGTTC (reverse) for human *GAPDH*; 5′-CCCTATGAGATCCGAGTCCA (forward) and 5′- CGTCCACATTTGTTGAGCAC (reverse) for human *NFASC-NTRK1*; 5′-ATGCGACCCTCCGGGACG (forward) and 5′-ATTCCGTTACACACTTTGCGGC (reverse) for human *EGFR* vIII.

### Western Blot

Total cell lysates were prepared in NP-40 lysis buffer (Invitrogen), with added protease inhibitor cocktail and 1 mM PMSF. The lysates were separated on SDS-PAGE and transferred to PVDF membranes. The following commercial antibodies were used: p-TrkA (Tyr674/675, Cell signaling), p-AKT (Ser473, Cell signaling), p-ERK (Thr202/Tyr204, Cell signaling), and β-actin (Sigma).

### Proliferation Assay

Proliferation assays were conducted with EZ-Cytox cell viability assay kit (Daeil Lab Service), according to the manufacturer's instruction. In detail, 10^3^ NIH 3T3 cells were seeded into 96-well plates (100 μl/well, DMEM +10% BCS). After 0, 1, or 3 days, 10 μl of EZ-Cytox reagent was added to each well and incubated for 2 hours. After incubation, light absorbance at wavelength 450 nm (foreground) and 650 nm (background) was measured using a spectrophotometer. For shRNA-mediated proliferation inhibition assays, the shRNA-infected cells were examined for proliferation at 0 or 2 days after plating. To calculate the percent inhibition mediated by each shRNA, the degree of proliferation during the 2-day period for each shRNA was compared to the corresponding degree for a non-specific, control shRNA.

### Soft Agar Assay

Soft agar colony formation assay was performed in 6-well plates. The base layer of each well consisted of 1 ml medium with a final concentration of 0.8% Noble agar (BD). After agar solidification, 10^4^ cells in 1 mL medium with 0.4% agar were seeded on the bottom agar layer and incubated for 30 days at 37°C and 5% CO_2_. After colonies were stained with 0.05% crystal violet (Sigma-Aldrich), they were microscopically observed (40X) and counted in randomly selected fields.

### Xenograft Tumor Formation Assay

Animal experiments were approved by the Institutional Review Boards of the Samsung Medical Center and conducted in accordance with the “National Institutes of Health Guide for the Care and Use of Laboratory Animals” (NIH publication 80–23). Nine-week-old male BALB/c nude mice (Orient Bio) were used for subcutaneous injection. 5×10^6^ cells were resuspended in Hank's Buffered Salt Solution (Invitrogen) and mixed with an equal volume of high concentration matrigel (BD Sciences). The mixture was injected into the right dorsal flank of mice. Swelling of the injected lesion over a diameter >200 mm^3^ was considered as a tumor incidence. Mice with the reduction of the total body weight by >20% were euthanized, and the masses at the injected sites were excised for histological confirmation of the tumors.

## Supporting Information

Figure S1 ***YEATS4-XRCC6BP1*** **fusion gene.**
(PDF)

Figure S2 **Expression of genes involved in** ***NTRK1*** **-fusion.** Expression of (**A**) *NTRK1*, (**B**) *NFASC*, and (**C**) *BCAN*, according to the human gene atlas. Neuronal tissues are indicated in red.
(PDF)

Figure S3 **Expression of** ***NFASC*** **and** ***BCAN*** **in normal brain and GBM.** Expression of (**A**) *NFASC*, and (**B**) *BCAN* in normal brain (N = 10) and GBM (N = 545, from 526 patients) samples of the TCGA panel. Expression was measured using Affymetrix Human Genome U133A arrays.
(PDF)

Figure S4 **Western blot analysis examining the potential downstream signaling molecules of the** ***NTRK1*** **fusion gene.**
(PDF)

Figure S5 **Genetic alterations in major GBM driver genes and pathways in the two TCGA GBM samples with the** ***NTRK1*** **fusion.**
(PDF)

Figure S6 **Domain structure of** ***NTRK1*** **and the two** ***NTRK1*** **fusion genes found in GBMs.**
(PDF)

Table S1 **Potential gene fusions predicted by FusionSeq.** For “Fusion Type”: intra, intra-chromosomal; inter, inter-chromosomal; read-through, the involved genes are adjacent to each other and are on the same strand; cis, the involved genes are adjacent and are on the opposite strands. “Inter-reads” is the number of discordant read pairs. “Intra-reads” is the total number of reads that map to the corresponding gene. SPER, DASPER and RESPER are FusionSeq-reported scores for prioritization. Candidates were sorted by RESPER (in descending order). DASPER >1.0 and RESPER >1.0 were used as cutoffs, as suggested by the authors of FusionSeq. Fusion events involving *NTRK1* are highlighted in yellow. Note that some entries (such as *LANCL2-SEPT14* for patient “0211”) are redundantly represented because RNA-Seq for some samples was done in replicates.
(XLSX)

Table S2 **Numbers of total RNA-Seq reads and predicted fusions in each sample.**
(XLSX)

Table S3 **NGF/TrkA (** ***NTRK1*** **) signature.**
(XLSX)
