# Correction: *NTRK1* Fusion in Glioblastoma Multiforme

**DOI:** 10.1371/journal.pone.0101140

**Published:** 2014-06-18

**Authors:** The PLOS ONE Staff

There is an error in the affiliation for the fifth author. Jaeyeol An is not affiliated with #4 but with #1 Samsung Biomedical Research Institute, Samsung Medical Center, Seoul, Korea and #2 Institute for Refractory Cancer Research, Samsung Medical Center, Seoul, Korea.

There is an error in the spelling of the third authors name in the PDF. The correct name is: Hee Jin Cho.
