# Supplementary figures and images for: NTRK1 Fusion in Glioblastoma Multiforme
Source: PLoS One. 2014 Mar 19;9(3):e91940. doi: 10.1371/journal.pone.0091940 (PMC3960150; doi:10.1371/journal.pone.0091940)

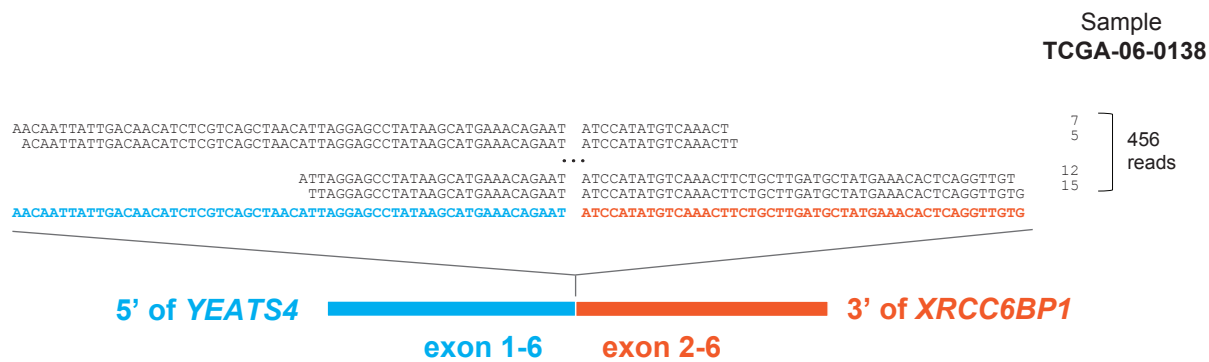

**Figure S1** *YEATS4-XRCC6BP1* fusion gene.

Supplement: Figure S1 — YEATS4-XRCC6BP1 fusion gene. (PDF) [file pone.0091940.s001.pdf]

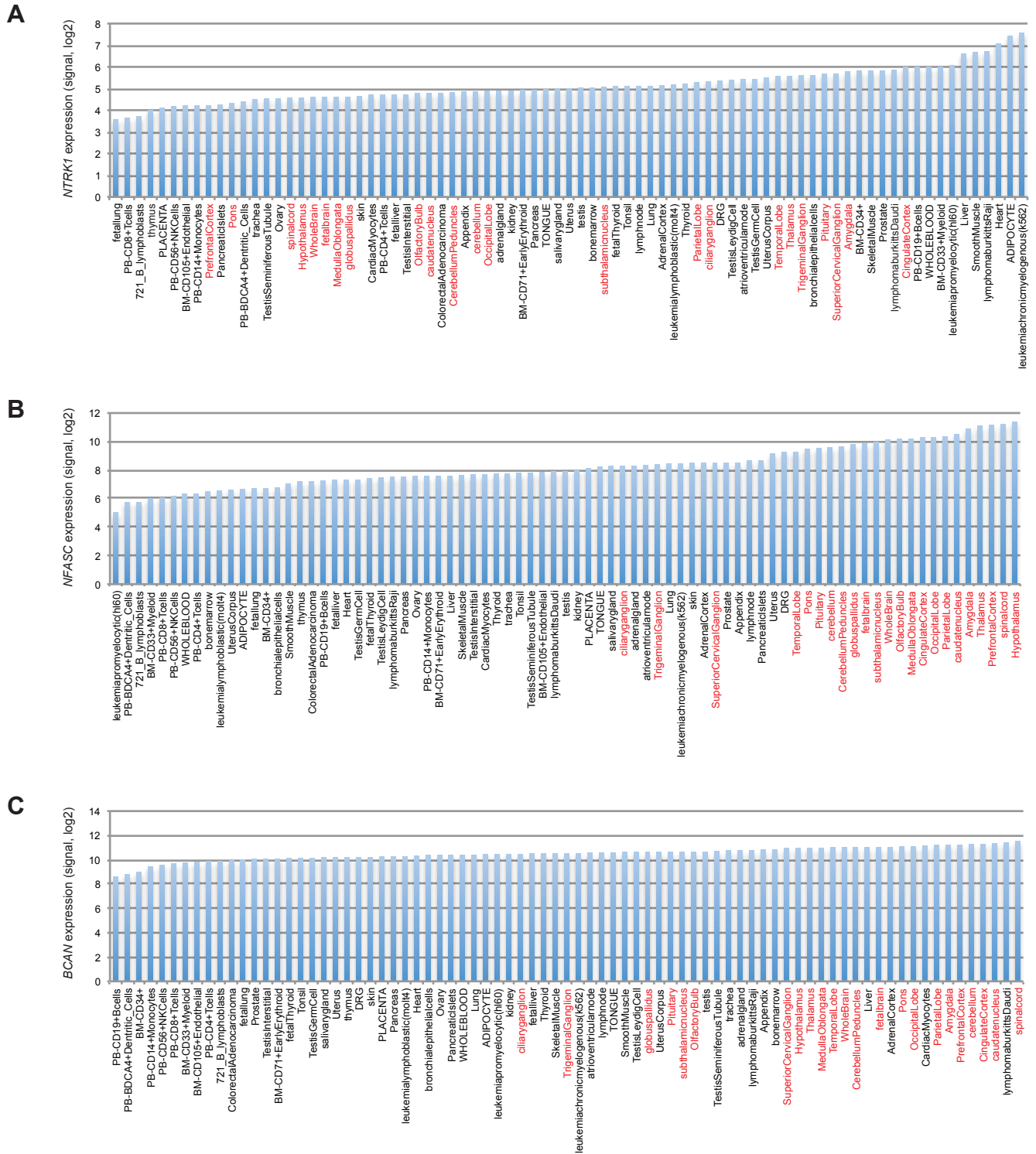

Supplement: Figure S2 — Expression of genes involved in NTRK1 -fusion. Expression of (A) NTRK1, (B) NFASC, and (C) BCAN, according to the human gene atlas. Neuronal tissues are indicated in red. (PDF) [file pone.0091940.s002.pdf]

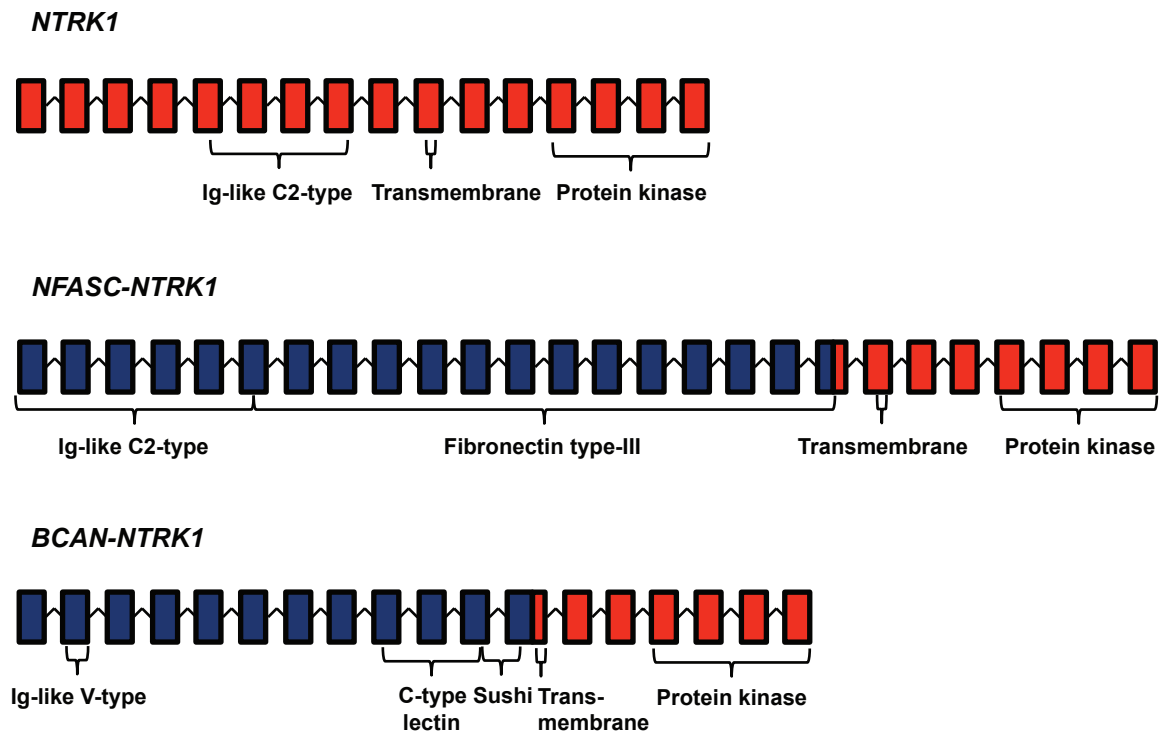

**Figure S6** Domain structure of *NTRK1* and the two *NTRK1* fusion genes found in GBM.

Supplement: Figure S6 — Domain structure of NTRK1 and the two NTRK1 fusion genes found in GBMs. (PDF) [file pone.0091940.s006.pdf]
