# Supplementary material for: NTRK1 Fusion in Glioblastoma Multiforme
Source: PLoS One. 2014 Mar 19;9(3):e91940. doi: 10.1371/journal.pone.0091940 (PMC3960150; doi:10.1371/journal.pone.0091940)
Supplement: Figure S3 — Expression of NFASC and BCAN in normal brain and GBM. Expression of (A) NFASC, and (B) BCAN in normal brain (N = 10) and GBM (N = 545, from 526 patients) samples of the TCGA panel. Expression was measured using Affymetrix Human Genome U133A arrays. (PDF) [file pone.0091940.s003.pdf]

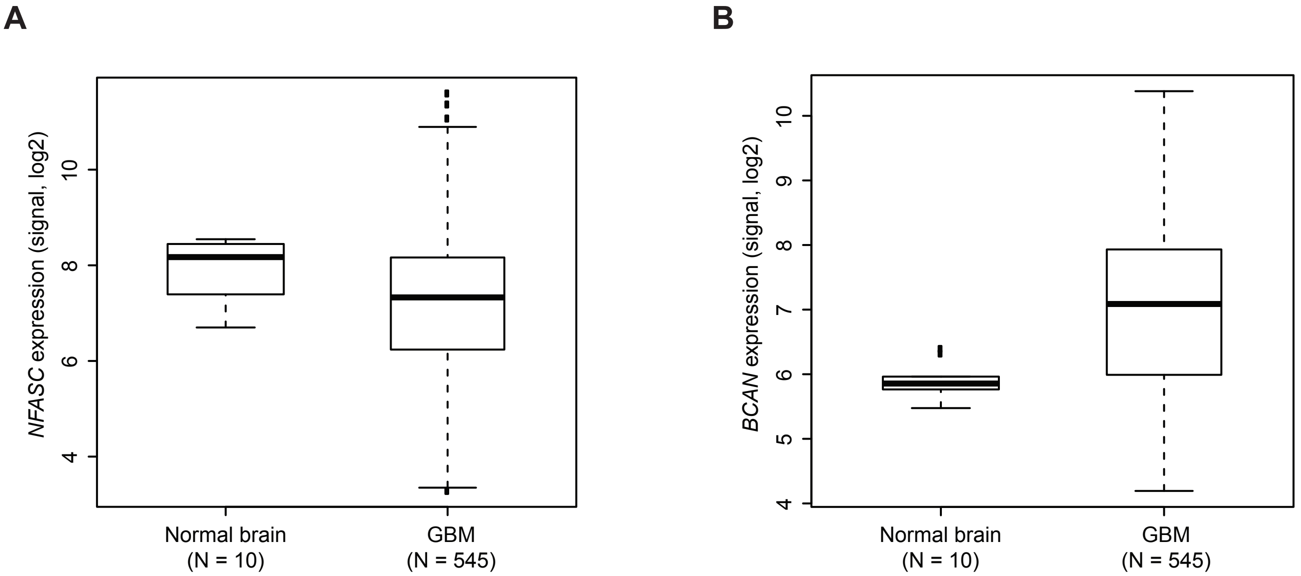

Figure S3 Expression of *NFASC* and *BCAN* in normal brain and GBM. Expression of (**A**) *NFASC*, and (**B**) *BCAN* in normal brain (N = 10) and GBM (N = 545, from 526 patients) samples of the TCGA panel. Expression was measured using Affymetrix Human Genome U133A arrays.
