# Supplementary material for: NTRK1 Fusion in Glioblastoma Multiforme
Source: PLoS One. 2014 Mar 19;9(3):e91940. doi: 10.1371/journal.pone.0091940 (PMC3960150; doi:10.1371/journal.pone.0091940)
Supplement: Figure S4 — Western blot analysis examining the potential downstream signaling molecules of the NTRK1 fusion gene. (PDF) [file pone.0091940.s004.pdf]

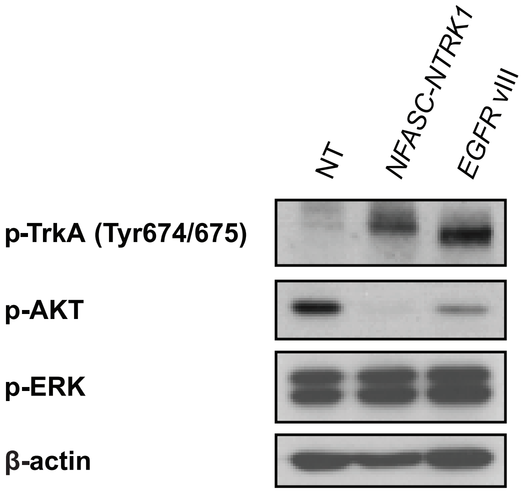

Figure S4 Western blot analysis examining the potential downstream signaling molecules of the *NTRK1* fusion gene.
