# Supplementary material for: NTRK1 Fusion in Glioblastoma Multiforme
Source: PLoS One. 2014 Mar 19;9(3):e91940. doi: 10.1371/journal.pone.0091940 (PMC3960150; doi:10.1371/journal.pone.0091940)
Supplement: Figure S5 — Genetic alterations in major GBM driver genes and pathways in the two TCGA GBM samples with the NTRK1 fusion. (PDF) [file pone.0091940.s005.pdf]

	TCGA-19-2619	TCGA-06-5411	
NTRK1	fusion with *BCAN*	fusion with *NFASC*	RTK family
EGFR	1.9 fold amplification	1.3 fold amplification	RTK family
PDGFRA	-	-	RTK family
MET	-	-	RTK family
FGFR3	-	-	RTK family
PIK3CA	-	-	PI3K pathway
PIK3R1	-	-	PI3K pathway
PTEN	-	nonsense mutation	PI3K pathway
AKT3	-	-	PI3K pathway
NF1	-	-	RAS pathway
BRAF	-	-	RAS pathway
TP53	-	-	p53 pathway
MDM2	-	-	p53 pathway
MDM4	-	7.0 fold amplification	p53 pathway
CDKN2A/B	homozygous deletion	homozygous deletion	p53 pathway; RB pathway
CDKN2C	-	homozygous deletion	RB pathway
CDK4	-	-	RB pathway
CDK6	-	-	RB pathway
CCND2	-	-	RB pathway
RB1	-	-	RB pathway
IDH1	-	-	

Figure S5 Genetic alterations in major GBM driver genes and pathways in the two TCGA GBM samples with *NTRK1* fusion.
